# Sex differences in direct healthcare costs following stroke: a population-based cohort study

**DOI:** 10.1186/s12913-021-06669-w

**Published:** 2021-06-29

**Authors:** Amy Y. X. Yu, Murray Krahn, Peter C. Austin, Mohammed Rashid, Jiming Fang, Joan Porter, Manav V. Vyas, Susan E. Bronskill, Eric E. Smith, Richard H. Swartz, Moira K. Kapral

**Affiliations:** 1 Department of Medicine (Neurology), University of Toronto, Sunnybrook Health Sciences Centre, 2075 Bayview Ave, Toronto, Ontario Canada; 2 ICES, Toronto, Ontario Canada; 3 Institute of Health Policy, Management, and Evaluation, University of Toronto, Toronto, Ontario Canada; 4 Department of Medicine (General Internal Medicine), University of Toronto-University Health Network, Toronto, Ontario Canada; 5 Toronto Health Economics and Technology Assessment, Toronto, Ontario Canada; 6 Department of Clinical Neurosciences, Community Health Sciences, and Hotchkiss Brain Institute, University of Calgary, Calgary, Alberta Canada

**Keywords:** Stroke, Sex, Healthcare cost, Health services research

## Abstract

**Background:**

The economic burden of stroke on the healthcare system has been previously described, but sex differences in healthcare costs have not been well characterized. We described the direct person-level healthcare cost in men and women as well as the various health settings in which costs were incurred following stroke.

**Methods:**

In this population-based cohort study of patients admitted to hospital with stroke between 2008 and 2017 in Ontario, Canada, we used linked administrative data to calculate direct person-level costs in Canadian dollars in the one-year following stroke. We used a generalized linear model with a gamma distribution and a log link function to compare costs in women and men with and without adjustment for baseline clinical differences. We also assessed for an interaction between age and sex using restricted cubic splines to model the association of age with costs.

**Results:**

We identified 101,252 patients (49% were women, median age [Q1-Q3] was 76 years [65–84]). Unadjusted costs following stroke were higher in women compared to men (mean ± standard deviation cost was $54,012 ± 54,766 for women versus $52,829 ± 59,955 for men, and median cost was $36,703 [$16,496–$72,227] for women versus $32,903 [$15,485–$66,007] for men). However, after adjustment, women had 3% lower costs compared to men (relative cost ratio and 95% confidence interval 0.97 [0.96,0.98]). The lower cost in women compared to men was most prominent among people aged over 85 years (p for interaction = 0.03). Women incurred lower costs than men in outpatient care and rehabilitation, but higher costs in complex continuing care, long-term care, and home care.

**Conclusions:**

Patterns of resource utilization and direct medical costs were different between men and women after stroke. Our findings inform public payers of the drivers of costs following stroke and suggest the need for sex-based cost-effectiveness evaluation of stroke interventions with consideration of costs in all care settings.

**Supplementary Information:**

The online version contains supplementary material available at 10.1186/s12913-021-06669-w.

## Introduction

Stroke is a leading cause of disability globally. In addition to affecting the lives of patients and their caregivers, stroke imposes a significant economic burden on the healthcare system [1, 2]. Compared to men, women have been found to have increased disability, poorer quality of life, and more frequent institutionalization after stroke [3, 4]. Poor functional outcome is associated with higher healthcare costs, [5] but sex differences in stroke-related healthcare costs are not well understood.

This information is important for several reasons. First, as the value of evaluating stroke care and outcomes stratified by sex is being increasingly recognized, [6, 7] information on cost by sex is necessary to inform cost-effectiveness studies of treatments and interventions. Second, identifying sex differences in cost may highlight areas of inequity or generate opportunities to reduce costs, which are relevant for policy makers and health system planning. Third, evaluating the settings in which the costs are incurred, whether they are in acute care, rehabilitation, outpatient services, or elsewhere, provides information on whether the drivers of costs are different for women and men. Further, this knowledge may help avoid the implementation of focused efforts to reduce cost in one setting (e.g. acute care) only to have these costs shifted to another setting (e.g. nursing homes) without any overall gain [8].

We described the direct healthcare cost in men and women as well as the healthcare settings in which these costs were incurred in the year after a hospitalization for stroke in Ontario, Canada with and without adjustment for comorbidities and costs incurred in the year prior to stroke.

## Methods

### Cohort identification and data sources

In this population-based retrospective cohort study, we identified all adults admitted to an acute care hospital with a most responsible diagnosis of ischemic stroke or intracerebral hemorrhage between April 1st 2008 and March 31st 2017 in Ontario, Canada’s most populous province with 14 million people [9, 10]. We excluded patients without valid health insurance numbers, and therefore not covered under the provincial health plan, and those with subarachnoid hemorrhage. Only the first hospitalization was included for patients with multiple admissions to create the cohort. We used administrative data to identify the study cohort, covariates, and costs. Supplemental Table 1 summarizes these datasets which have been validated and extensively used for research, and are housed at ICES (previously the Institute for Clinical Evaluative Sciences) [11, 12]. The datasets were linked using unique encoded identifiers and analyzed at ICES.

### Outcomes

The primary outcome was the direct person-level cumulative cost, calculated from the perspective of the government payer in the first 365 days after the date of the index hospital admission. We estimated the costs of services borne by the Ontario Ministry of Health and Long-Term Care, [13] the public payer in Ontario, including 1) acute care, including emergency department visits and hospitalizations for the index event as well as subsequent health encounters in acute care, 2) outpatient care, including laboratory tests, physician services, and prescription drugs for patients aged 65 and over, 3) inpatient rehabilitation, 4) publicly-funded home care, which may range from a few hours a week to a few hours a day for community-dwelling people who require assistance with activities of daily living or instrumental activities of daily living, 5) complex continuing care, which provides care to patients who have long-term illnesses or disabilities that require skilled care not available in long-term care facilities, and 6) long-term care, which are facilities for adults who need continuous nursing care and assistance with activities of daily living.

Direct costs to the Ontario public payer of physician billing for health services, prescription drugs, and outpatient diagnostic or laboratory services were calculated for each patient. Home care costs were estimated using the average cost per hour. Long-term care costs were calculated based on the government’s per diem payment rate. Given costs of encounters in the emergency department, hospital, and complex continuing care settings depend on the intensity of resource utilization, each encounter in these settings was assigned a resource intensity weight associated with its case-mix group, allowing for the calculation of the weighted cost for each visit based on the intensity of use of drugs, procedures, tests, and personnel. Resource intensity weights were calculated using standard methods from the Canadian Institute for Health Information [14]. Our methods follow Ontario’s guidelines on person-level costing calculations using administrative data [13]. Costs were adjusted to 2018 Canadian dollars using the Statistics Canada Consumer Price Index for Health [15].

### Statistical methods

We used a generalized linear model with a gamma distribution and a log link function to assess the association between sex and one-year costs following stroke with and without adjustment for covariates [16]. The interpretation of the exponential of the estimated regression coefficient, which we refer to as a relative cost ratio, is the relative change in mean cost associated with a one-unit change in the covariate for continuous variables or relative to a reference group for categorical variables. Covariates were selected based on clinical relevance and included pre-stroke one-year direct healthcare costs (continuous), age category, home location (rural versus urban), neighbourhood-level income quintile, stroke type (ischemic versus hemorrhagic) [10], vascular comorbidity (diabetes, hypertension, dyslipidemia, atrial fibrillation, prior stroke, coronary artery disease, peripheral vascular disease) [17–20], frailty using the hospital frailty risk score [21], and stroke severity using the Passive Surveillance Stroke Severity indicator [22]. Home location and neighbourhood income quintile were obtained from the Canadian census and Postal Code Conversion File [23]. Given the healthcare system incurs no additional costs after death, we showed crude mean costs for women and men stratified by one-year mortality status as well as the unadjusted and adjusted relative cost ratio comparing women to men. In addition, we assessed whether there was an interaction between age and sex by using restricted cubic splines with five knots. In that model, we included an interaction between age as a continuous variable and sex so that the effect of sex on costs will be allowed to vary across the age spectrum. All data analyses were performed using SAS version 9.4 (SAS Institute Inc., Cary, North Carolina).

## Results

We identified 101,252 patients, of whom 49% were women. Baseline characteristics are described in Table 1. Women tended to be older than men at the time of stroke, were more likely to have comorbid hypertension, atrial fibrillation, and to have higher frailty and stroke severity scores; while men were more likely to have coronary artery disease, diabetes, and peripheral artery disease than women. In the one-year prior to stroke, women had higher unadjusted healthcare costs compared to men (mean ± standard deviation cost of $15,956 ± 27,928 for women versus $12,827 ± 26,589 for men).

**Table 1:** Baseline characteristics by sex

	Women ***n*** = 49,419	Men ***n*** = 51,833	***P***-value
**Age**			
Median (interquartile range)	79 (69–86)	72 (62–81)	<.001
**Age categories**			<.001
18–45 years	1586 (3.2%)	1959 (3.8%)	
46–65 years	8278 (16.8%)	15,214 (29.4%)	
66–75 years	9515 (19.3%)	13,025 (25.1%)	
76–85 years	16,274 (32.9%)	14,798 (28.5%)	
> 85 years	13,766 (27.9%)	6837 (13.2%)	
**Hypertension**	41,968 (84.9%)	41,475 (80.0%)	<.001
**Diabetes**	17,032 (34.5%)	19,843 (38.3%)	<.001
**Atrial fibrillation**	6117 (12.4%)	4823 (9.3%)	<.001
**Dyslipidemia**	14,742 (29.8%)	15,346 (29.6%)	0.436
**History of stroke**	3747 (7.6%)	4061 (7.8%)	0.132
**Coronary artery disease**	7045 (14.3%)	11,281 (21.8%)	<.001
**Peripheral vascular disease**	2154 (4.4%)	3762 (7.3%)	<.001
**Frailty score**			
Low frailty (score < 5)	21,514 (43.5%)	27,412 (52.9%)	<.001
Intermediate frailty (score 5–15)	19,565 (39.6%)	18,722 (36.1%)	
High frailty (> 15)	8340 (16.9%)	5699 (11.0%)	
**Income quintile**			<.001
Highest	8279 (16.8%)	9073 (17.5%)	
Second to highest	8694 (17.6%)	9478 (18.3%)	
Middle	9514 (19.3%)	10,096 (19.5%)	
Second to lowest	10,910 (22.1%)	11,116 (21.4%)	
Lowest	12,022 (24.3%)	12,070 (23.3%)	
**Rural residence**	6019 (12.2%)	7051 (13.6%)	<.001
**Stroke type**			0.048
Ischemic stroke	43,052 (87.1%)	44,938 (86.7%)	
Intracerebral hemorrhage	6367 (12.9%)	6895 (13.3%)	
**Stroke severity**			<.001
Mild stroke	19,672 (39.8%)	29,485 (56.9%)	
Moderate stroke	27,002 (54.6%)	19,917 (38.4%)	
Severe stroke	2745 (5.6%)	2431 (4.7%)	

**Fig. 1:**
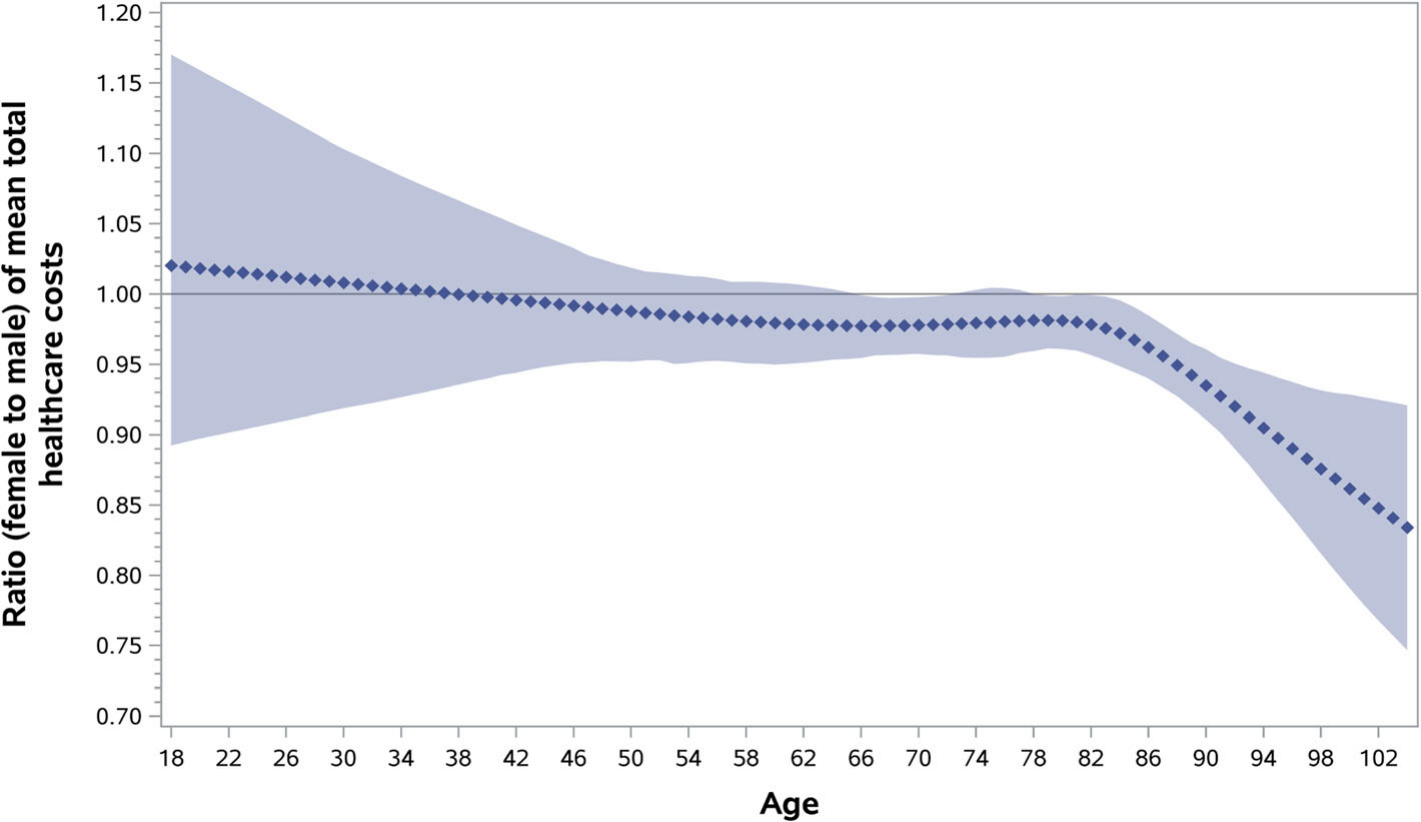
Adjusted relative cost ratio of mean total healthcare costs comparing female to male by age (shaded area represents confidence band)

Table 2 shows that costs following stroke were high and remained higher for women than men (mean ± standard deviation cost of $54,012 ± 54,766 for women versus $52,829 ± 59,955 for men, and median (Q1-Q3) cost was $36,703 ($16,496–$72,227) for women versus $32,903 ($15,485–$66,007) for men, distribution of costs can be found in Supplemental Figure 1). After adjusting for covariates, the mean costs following stroke were 3% lower in women compared to men (relative cost ratio and 95% confidence interval 0.97 [0.96,0.98]). We found a statistically significant interaction between age and sex (Wald test, p for interaction = 0.03) where the reduced cost in women compared to men is most prominent in patients above age 85 (Fig. 1).

**Table 2:** Mean ± SD direct healthcare costs in the one-year following stroke and relative cost ratio [95% CI] comparing women to men

	Mean cost ± SD Women	Mean cost ± SD Men	Unadjusted relative cost ratio^a^ [95% CI]	Adjusted relative cost ratio^b^ [95% CI]
**All patients** **(*n*** **= 101,252)**	$54,012 ± $54,766	$52,829 ± $59,955	1.02 [1.01, 1.04]	0.97 [0.96, 0.98]
**Patients who died within 1 year (*n*** **= 27,252)**	$40,152 ± $46,541	$46,673 ± $57,382	0.86 [0.83, 0.88]	0.90 [0.88, 0.92]
**Patients who were alive at 1 year** **(*n*** **= 74,000)**	$60,000 ± $56,926	$54,753 ± $60,610	1.10 [1.08, 1.11]	0.98 [0.97, 0.99]

*SD* Standard deviation, *CI* Confidence interval.

^a^Exponentiated regression coefficient of the generalized linear model with a gamma distribution and a log link function

^b^Adjusted for age, income quintile, rurality, hypertension, diabetes, atrial fibrillation, dyslipidemia, history of stroke, coronary artery disease, peripheral vascular disease, frailty, stroke type, stroke severity, and one-year pre-stroke cost (continuous)

**Fig. 2:**
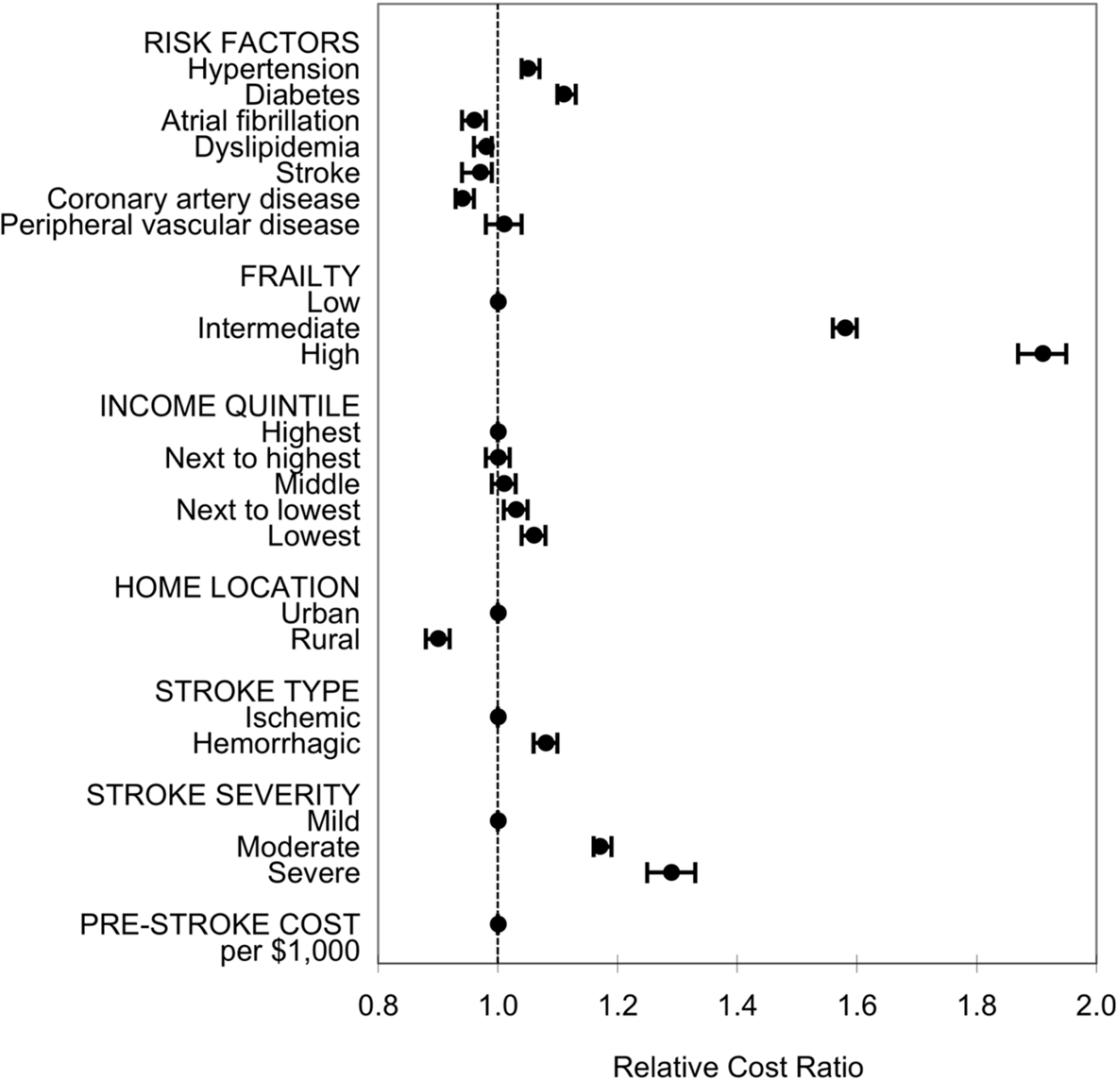
Relative change in direct one-year healthcare costs associated with the covariates

Figure 2 shows the association between cost and all the covariates. We found that moderate and severe stroke (versus mild stroke), intracerebral hemorrhage (versus ischemia), and higher baseline frailty (versus lower frailty) were associated with increased cost, while rural residence was associated with lower cost compared to those living in non-rural areas. Living in the lowest and second to lowest neighborhood-level income quintiles was associated with higher cost compared to living in the highest income quintile neighborhood.

The relative cost ratio is the exponentiated regression coefficient of the generalized linear model with a gamma distribution and a log link function. The coefficients for each covariate are shown in Supplemental Table 5. Given the interaction between sex and age, hazard ratio for sex across the full spectrum of age is shown in Fig. 1.

A higher proportion of women than men died within 1 year (30% versus 24%, *p* < 0.001), but the hazard of death in the two groups was similar after adjustment for all covariates (hazard ratio and 95% confidence interval 0.98 [0.95,1.00], Supplemental Figure 2). Stratified by mortality status at one-year, direct unadjusted costs were lower in women than men among patients who died, and costs were higher in women among those who survived (Table 2). After adjusting for covariates, costs were 10% lower in women than men among the patients who died in the first year and 2% lower in women among those who survived. After accounting for the interaction between age and sex, costs were lower in women compared to men between the ages of 72 and 88 years among those who died within the first year (p for interaction = 0.02, Supplemental Figure 3), and costs were lower in women compared to men between the ages of 50 and 55 years as well as above 86 years among those who survived the full year (p for interaction < 0.001, Supplemental Figure 4).

**Fig. 3:**
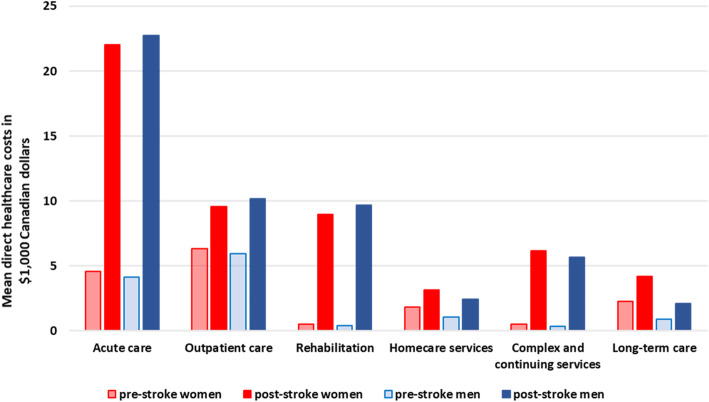
Mean direct healthcare costs in the year prior and following stroke by healthcare settings and sex

Acute care accounted for most of the costs in the first year for both groups (Fig. 3). Costs in outpatient and rehabilitation services were lower in women than men, and costs in home care, complex continuing care and long-term care were higher in women, but the absolute differences were small (Supplemental Table 3). Among patients who died within the first year, the sex difference in cost was more prominent in acute care, outpatient care, and rehabilitation; however, among those who survived, the difference was more prominent in the settings of home care, complex continuing care, and long-term care (Supplemental Table 4).

## Discussion

In this large population-based cohort of patients admitted to acute care hospital with stroke, we found that healthcare costs were high, with a mean per-person cost of over $50,000, which is higher than the mean cost of caring for people in the first year after diagnosis of cancer ($25,914) [24] or coronary artery disease ($23,000-26,000) [25] in Canada. While unadjusted costs were higher in women than men, the adjusted costs were lower in women.

Differences in baseline characteristics between men and women likely accounted for some of the cost differences as the magnitude and direction of sex difference in costs changed after adjustment for baseline characteristics. We expected that the direct healthcare costs from the public government payer’s perspective would be lower in the patients with shorter survival after stroke, compared to those who survive longer. We showed that costs were lower in women compared to men regardless of survival. Costs were 2% lower in women than men among patients alive at 1 year and 10% lower in women among those who died (Table 2). Therefore, sex differences in direct healthcare costs are not fully explained by potential differences in survival. Dedicated work on sex differences in costs by survival and accounting for potential changes in healthcare costs in the period preceding death is needed.

We found an interaction between age and sex. Costs were lower for women compared to men in patients above the age of 45 years, but this difference was most prominent for those above age 85 years. In the stratified analysis by mortality, the lower costs in women compared to men were also seen in younger age groups. It is not clear if the lower healthcare expenditures in women compared to men reflect dissimilarities in healthcare needs after stroke, quality of care, or other factors not accounted for in the current study, such as disparities in social or financial resources (e.g., having access to family member caregivers or the ability to hire private support), that could result in differential reliance on publicly funded care. Future work on the reasons for sex differences in cost with information on quality indicators of care is needed.

Our findings of differences in healthcare utilization patterns highlight the importance of considering all care settings when evaluating the cost-effectiveness of stroke interventions in men and women. For example, a treatment that reduces the need for long-term care may have more impact on reducing costs in women than men. Our work also informs the planning of bundled payment models to ensure that care needs are appropriately reimbursed and shifts in costs from one health setting to another are avoided [26, 27].

In addition, we reported on different predictors of costs following stroke. Higher stroke severity, frailty, and intracerebral hemorrhage were associated with higher costs, which is consistent with prior work and our understanding of the disease [5, 28]. We also found that rural location and neighborhood income quintiles, but not baseline healthcare costs, were associated with costs following stroke. These findings require further work to confirm and evaluate the possible causes. Finally, the high cost of stroke care reported in the current study is consistent with prior work on the economic burden of stroke [5, 29]. Recent work has shown reduction in stroke incidence in men, but not in women, among people aged over 85 years as well as a worrisome increase in stroke incidence among men under the age of 45 years [30]. Our findings call for renewed efforts for stroke prevention in all patient groups.

The current study has several strengths. Canada’s single-payer universal healthcare allowed us to examine individual-level direct costs in different healthcare settings that reflect the continuum of stroke care. We also considered pre-stroke costs which are not routinely included in health economic evaluations of stroke, [29] but this context is important because women in our study were older, had higher frailty, and higher baseline costs compared to men.

There are nevertheless several limitations worth discussing. First, we evaluated cost from the perspective of the Ontario public healthcare payer and could not account for costs incurred by individuals and their families, private insurers, nor societal costs such as income loss or other opportunity costs [31]. Second, we did not have information on the use of thrombolytics or endovascular thrombectomy, which is relevant because sex differences in the use of revascularization treatments and outcomes after treatment have been described [32, 33]. Third, we only evaluated drug-related costs in patients aged 65 years and older, as public healthcare only covers drug costs in this population. Nevertheless, prescription drugs only account for a small proportion of healthcare costs. Residual confounding could have affected the results despite adjustment for a wide range of baseline characteristics; alternative modeling approaches such as propensity-matched analyses or analysis of differences in costs using each patient as their own control could be explored in future work. Fourth, the generalizability of our findings to other jurisdictions without universal access to a central government-funded health care system is limited. Finally, we did not have information on patient functional status and therefore could not study cost in the context of disability.

## Conclusion

We quantified the sex differences in healthcare costs of stroke and found the settings where these costs were incurred were different between men and women. Future research on the mediators of cost differences in women and men after stroke, including how much of the costs are related to differences in post-stroke mortality and disability, is needed to understand the efficiency of healthcare expenditures and to optimise patient outcomes.

## Supplementary Information

**Additional file 1.**

## Data Availability

The data that support the findings of this study are available from ICES but restrictions apply to the availability of these data, which were used under license for the current study, and so are not publicly available. Data are however available from the authors upon reasonable request and with permission of ICES.
