# Supplementary material for: Sex differences in direct healthcare costs following stroke: a population-based cohort study
Source: BMC Health Serv Res. 2021 Jun 29;21:619. doi: 10.1186/s12913-021-06669-w (PMC8240191; doi:10.1186/s12913-021-06669-w)

**SUPPLEMENTARY MATERIAL**

**Sex differences in direct healthcare costs following stroke: A population-based cohort study**

Amy Y X Yu MD MSc^1,2,3^, Murray Krahn MD MSc^2,3,4,5^, Peter C Austin PhD^2,3^, Mohammed Rashid MSc^2^, Jiming Fang PhD^2^, Joan Porter MSc^2^, Manav V Vyas MBBS MSc^1,2,3^, Susan E Bronskill PhD^2,3^, Eric E Smith MD MPH^6^, Richard H. Swartz MD PhD^1,2^, Moira K Kapral MD MSc^2,3,4^

^1^Department of Medicine (Neurology), University of Toronto, Sunnybrook Health Sciences Centre, Toronto, Ontario, Canada, ^2^ICES, Toronto, Ontario, Canada, ^3^Institute of Health Policy, Management, and Evaluation, University of Toronto, ^4^Department of Medicine (General Internal Medicine), University of Toronto-University Health Network, Toronto, Ontario, Canada, ^5^Toronto Health Economics and Technology Assessment, Toronto, Ontario, Canada, ^6^Department of Clinical Neurosciences, Community Health Sciences, and Hotchkiss Brain Institute, University of Calgary, Calgary, Alberta, Canada.

Supplemental Table I Administrative health data sources

| **Database** | **Full database name** | **Description** |
| --- | --- | --- |
| **DAD** | Discharge Abstract Database | Inpatient hospitalization data |
| **NACRS** | National Ambulatory Care Reporting System | Emergency department data |
| **NRS** | National Rehabilitation Reporting System | Rehabilitation data |
| **CCRS** | Continuing Care Reporting System | Complex continuing care |
| **CCRS-LTC** | Continuing Care Reporting System Long-Term Care | Long-term care data |
| **HCD** | Ontario home care database | Home-care data |
| **OHIP** | Ontario Health Insurance Plan | Outpatient physician billings data |
| **ODBP** | Ontario Drug Benefit Program | Pharmacy data |
| **NDFP** | New Drug Funding Program | Chemotherapy drugs |
| **RPDB** | Ontario Registered Persons Database | Mortality data |

Supplemental Table II Case definitions for covariates

| **Covariate** | **Database** | **Case definition** |
| --- | --- | --- |
| **Age** | Registered Persons Database | Age |
| **Stroke type** | Discharge Abstract Database | Ischemic stroke: H34.1, I63.x, I64.x  Intracerebral hemorrhage: I61.x |
| **Diabetes** | Discharge Abstract Database  Ontario Health Insurance Plan  Ontario Drug Benefit Program | ≥2 outpatient claims (ICD-9 250) in a one-year period  or  ≥1 hospitalization (ICD-10-CA E10, E11, E13, E14)  or  ≥1 diabetes drug claim in a one-year period |
| **Hypertension** | Discharge Abstract Database  Ontario Health Insurance Plan | ≥1 hospitalization (ICD-10-CA I10.x, I11.x, I12.x, I13.x, or I15.x) or  ≥2 outpatient claims (ICD-9 401.x, 402.x, 403.x, 404.x, or 405.x) in a two-year period  or  1 outpatient claim followed by 1 outpatient/hospitalization within two years |
| **Dyslipidemia** | Ontario Health Insurance Plan | 2 outpatient claims (ICD-9 272) in 2 years |
| **Atrial fibrillation** | Discharge Abstract Database  Ontario Health Insurance Plan  National Ambulatory Care Reporting System | 1 hospitalization or 1 emergency department visit (ICD-10-CA I48)  or  4 outpatient claims (ICD-9 427) in a 1-year period |
| **Coronary artery disease** | Discharge Abstract Database  Same Day Surgery Database | ICD10-CA I21, I22  or  CCI 1IJ50, 1IJ57GQ, 1IJ54, or 1IJ76 |
| **Peripheral vascular disease** | Discharge Abstract Database  Same Day Surgery Database | ICD-10-CA I713, I714, I702, I739, I743, or I744  or  CCI 1JE57, 1JE50, or 1JE87 |

ICD-9 International classification of diseases, ninth revision, ICD-10-CA – International classification of diseases, tenth revision, Canada, CCI – Canadian Classification of Health Interventions

Supplemental Table III Mean ± standard deviation of direct healthcare costs in the one-year pre-stroke, the one-year post-stroke, and the differences in cost (post-stroke minus pre-stroke) by sex

|  | **Female**  **n=49,419** | | **Male**  **n=51,833** | | **p-value** |
| --- | --- | --- | --- | --- | --- |
|  | **Pre-stroke** | **Post-stroke** | **Pre-stroke** | **Post-stroke** |  |
| **Acute care** | 4,557 ± 14,380 | 22,033 ± 30,811 | 4,138 ± 14,010 | 22,745 ± 36,868 |  |
| Mean difference in cost | 17,476 ± 32,903 | | 18,606 ± 38,330 | | <0.001 |
| **Outpatient care** | 6,333 ± 11,883 | 9,547 ± 10,560 | 5,968 ± 12,155 | 10,195 ± 11,570 |  |
| Mean difference in cost | 3,214 ± 11,749 | | 4,227 ± 12,333 | | <0.001 |
| **Rehabilitation** | 504 ±  3,657 | 8,964 ± 15,764 | 403 ±  3,640 | 9,698 ± 16,525 |  |
| Mean difference in cost | 8,460 ± 16,163 | | 9,295 ± 16,874 | | <0.001 |
| **Homecare services** | 1,812 ± 5,244 | 3,140 ± 7,134 | 1,051 ± 4,120 | 2,411 ± 6,092 |  |
| Mean difference in cost | 1,328 ± 7,708 | | 1,361 ± 6,456 | | 0.459 |
| **Complex and continuing services** | 484 ±  6,220 | 6,152 ± 24,823 | 350 ±  5,805 | 5,668 ± 24,725 |  |
| Mean difference in cost | 5,667 ± 25,166 | | 5,319 ± 24,910 | | 0.027 |
| **Long-term care** | 2,265 ± 9,247 | 4,176 ± 11,551 | 917 ±  5,889 | 2,111 ± 8,098 |  |
| Mean difference in cost | 1,911 ± 12,252 | | 1,194 ± 8,232 | | <0.001 |
| **Total cost** | 15,956 ± 27,928 | 54,012 ± 54,766 | 12,827 ± 26,589 | 52,829 ± 59,955 |  |
| Mean difference in cost | 38,056 ± 58,774 | | 40,002 ± 62,328 | | <0.001 |

Supplemental Table IV Mean ± standard deviation of direct healthcare costs in the one-year post-stroke by sex and by survival.

|  | **Died within 1 year**  **n=27,252** | | **Alive at 1 year**  **n=74,000** | |
| --- | --- | --- | --- | --- |
|  | **Women**  **n=14,910** | **Men**  **n=12,342** | **Women**  **n=34,509** | **Men**  **n=39,491** |
| **Acute care** | 23,802 ± 31,593 | 28,417 ± 41,491 | 21,269 ± 30,436 | 20,972 ± 35,113 |
| **Outpatient care** | 5,958 ± 7,852 | 7,284 ± 9,513 | 11,098 ± 11,185 | 11,105 ± 11,998 |
| **Rehabilitation** | 3,385 ± 10,393 | 4,382 ± 12,037 | 11,374 ± 17,028 | 11,359 ± 17,365 |
| **Homecare services** | 1,475 ± 4,946 | 1,345 ± 4,276 | 3,859 ± 7,784 | 2,745 ± 6,522 |
| **Complex and continuing services** | 3,617 ± 15,510 | 4,036 ± 17,345 | 7,247 ± 27,830 | 6,178 ± 26,595 |
| **Long-term care** | 1,915 ± 6,495 | 1,210 ± 5,232 | 5,153 ± 13,026 | 2,393 ± 8,785 |
| **Total cost** | 40,152 ± 46,541 | 46,673 ± 57,382 | 60,000 ± 56,926 | 54,753 ± 60,610 |

Supplemental Table V Regression coefficients for each covariate of the generalized linear model with a gamma distribution and a log link function

| **Variable** | **Regression coefficient** |
| --- | --- |
| Intercept | 10.3394 |
| sex=F | 0.0384 |
| age † | 0.0010 |
| age' † | 0.0066 |
| age'' † | -0.0498 |
| age''' † | -0.0080 |
| sex=F * age † | -0.0010 |
| sex=F * age' † | 0.0019 |
| sex=F * age'' † | -0.0049 |
| sex=F * age''' † | -0.0480 |
| Hypertension | 0.0523 |
| Diabetes | 0.1052 |
| Atrial fibrillation | -0.0427 |
| Dyslipidemia | -0.0239 |
| History of stroke | -0.0347 |
| Coronary artery disease | -0.0584 |
| Peripheral vascular disease | 0.0083 |
| Intermediate frailty (score 5-15) | 0.4584 |
| High frailty (>15) | 0.6485 |
| Income quintile |  |
| Second to highest | 0.0025 |
| Middle | 0.0074 |
| Second to lowest | 0.0282 |
| Lowest | 0.0612 |
| Rural residence | -0.1052 |
| Intracerebral hemorrhage | 0.0727 |
| Moderate stroke | 0.1579 |
| Severe stroke | 0.2537 |
| Pre-stroke cost (per $1,000) | 0.0012 |

† Represent the component terms in the restricted cubic spline model that accounts for the interaction between age and sex

Supplemental Figure I Distribution of the overall direct healthcare cost in the year following stroke by sex


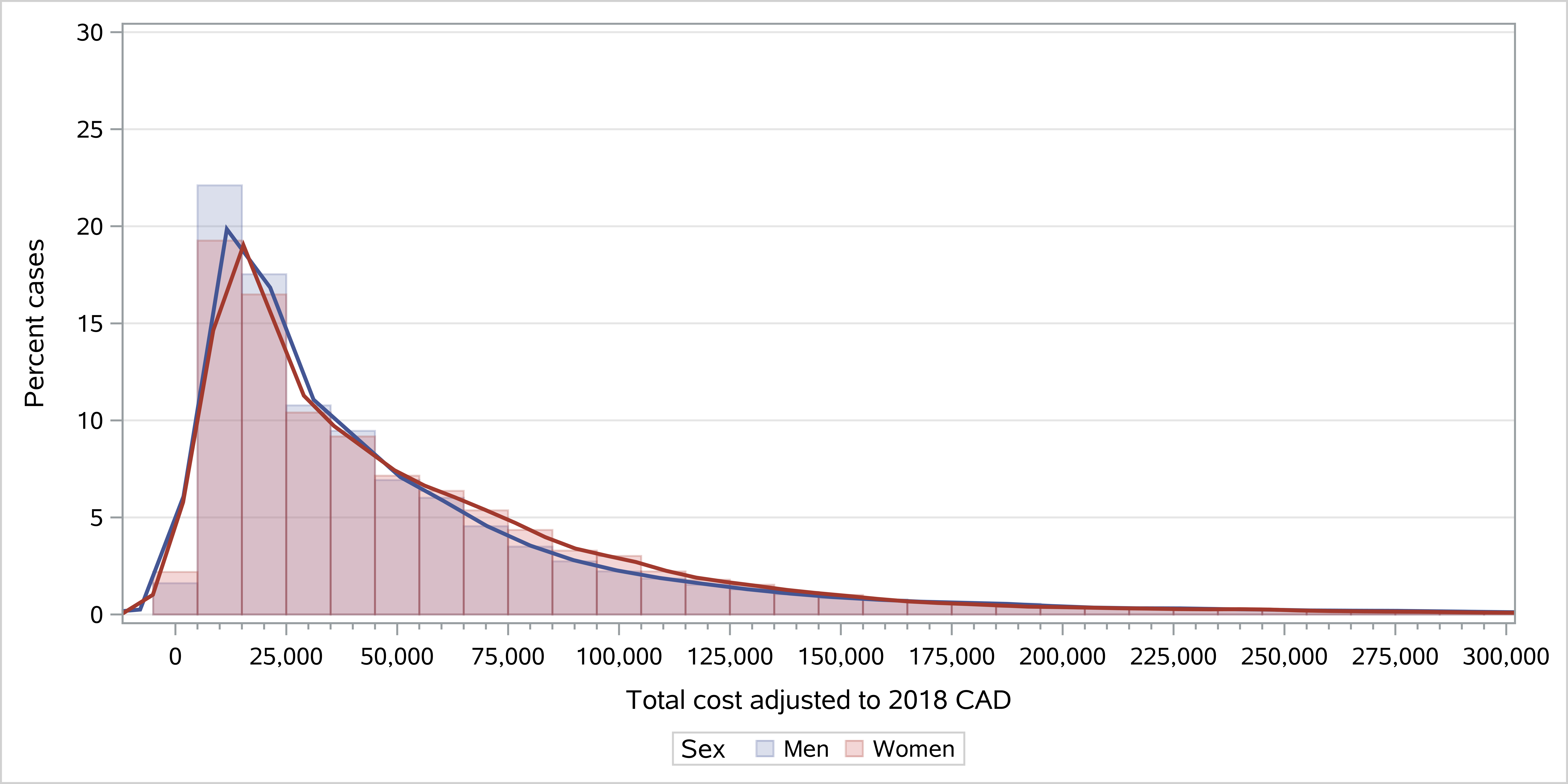


Supplemental Figure II Adjusted one-year survival stratified by sex from the proportional hazard models


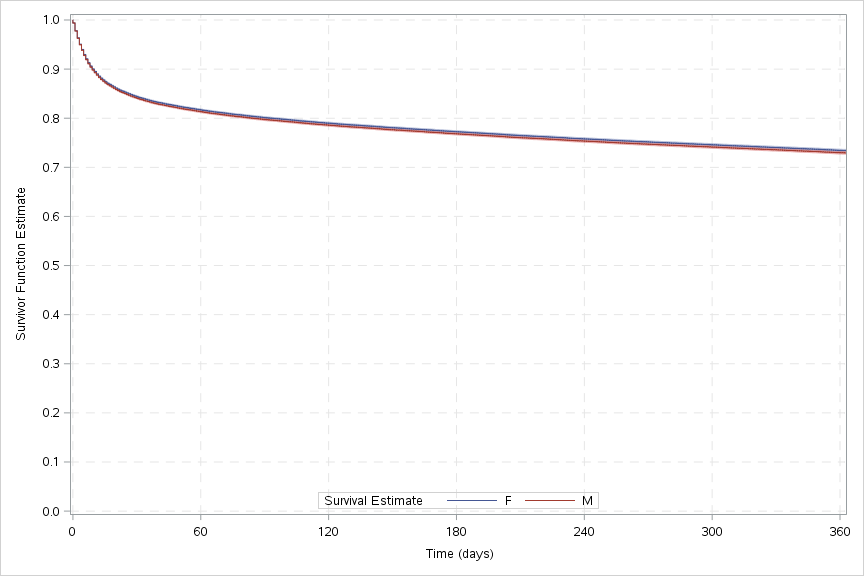


Adjusted hazard ratio and 95% confidence intervals for one-year all-cause mortality: 0.98 [0.95,1.00]. Adjusted for age, income quintile, rurality, hypertension, diabetes, atrial fibrillation, dyslipidemia, history of stroke, coronary artery disease, peripheral vascular disease, frailty, stroke type, and stroke severity

Supplemental Figure III Adjusted relative cost ratio of mean total healthcare costs comparing female to male by age (shaded area represents confidence band) among people who died within the first year


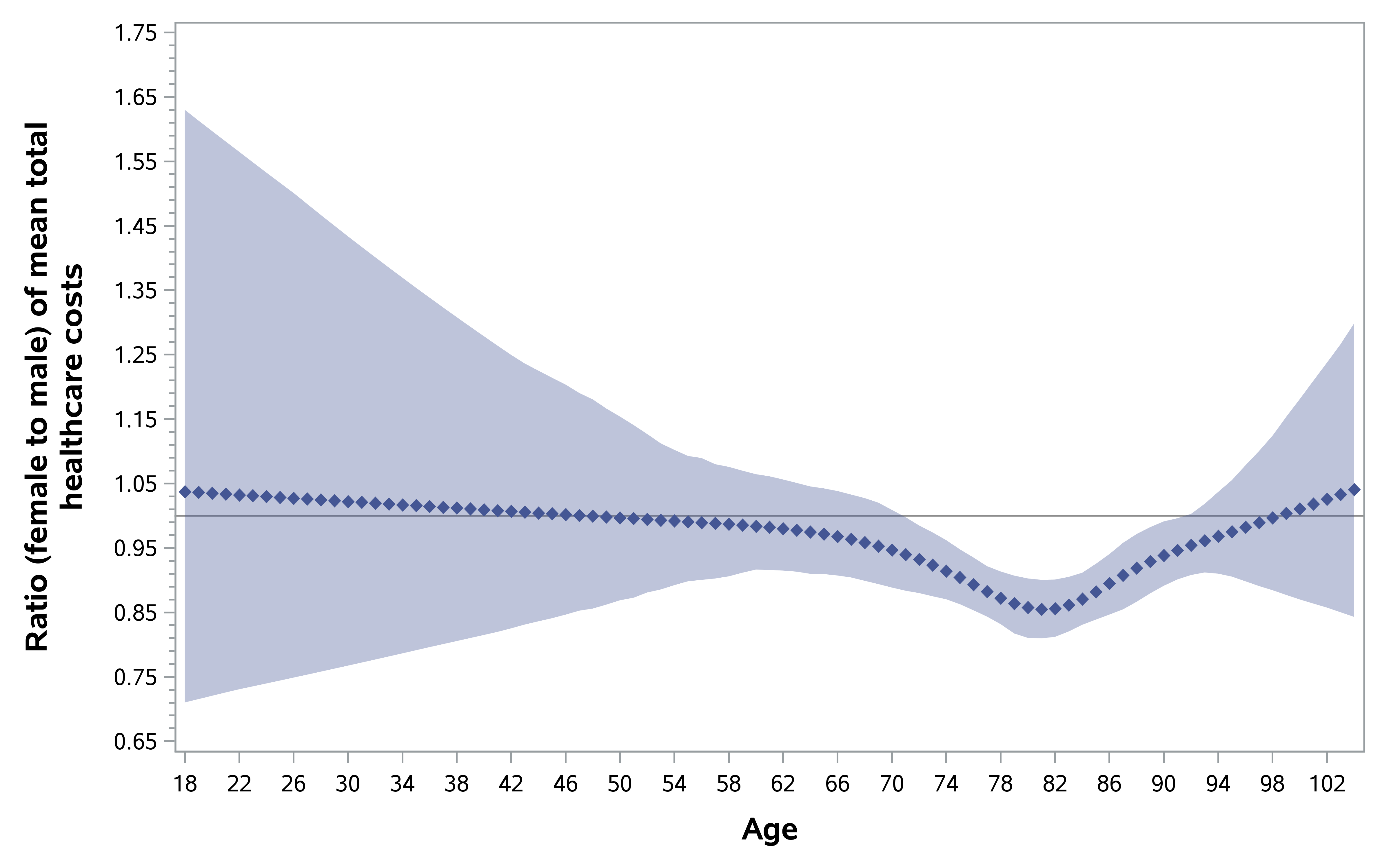


Supplemental Figure IV Adjusted relative cost ratio of mean total healthcare costs comparing female to male by age (shaded area represents confidence band) among people who survived the first year


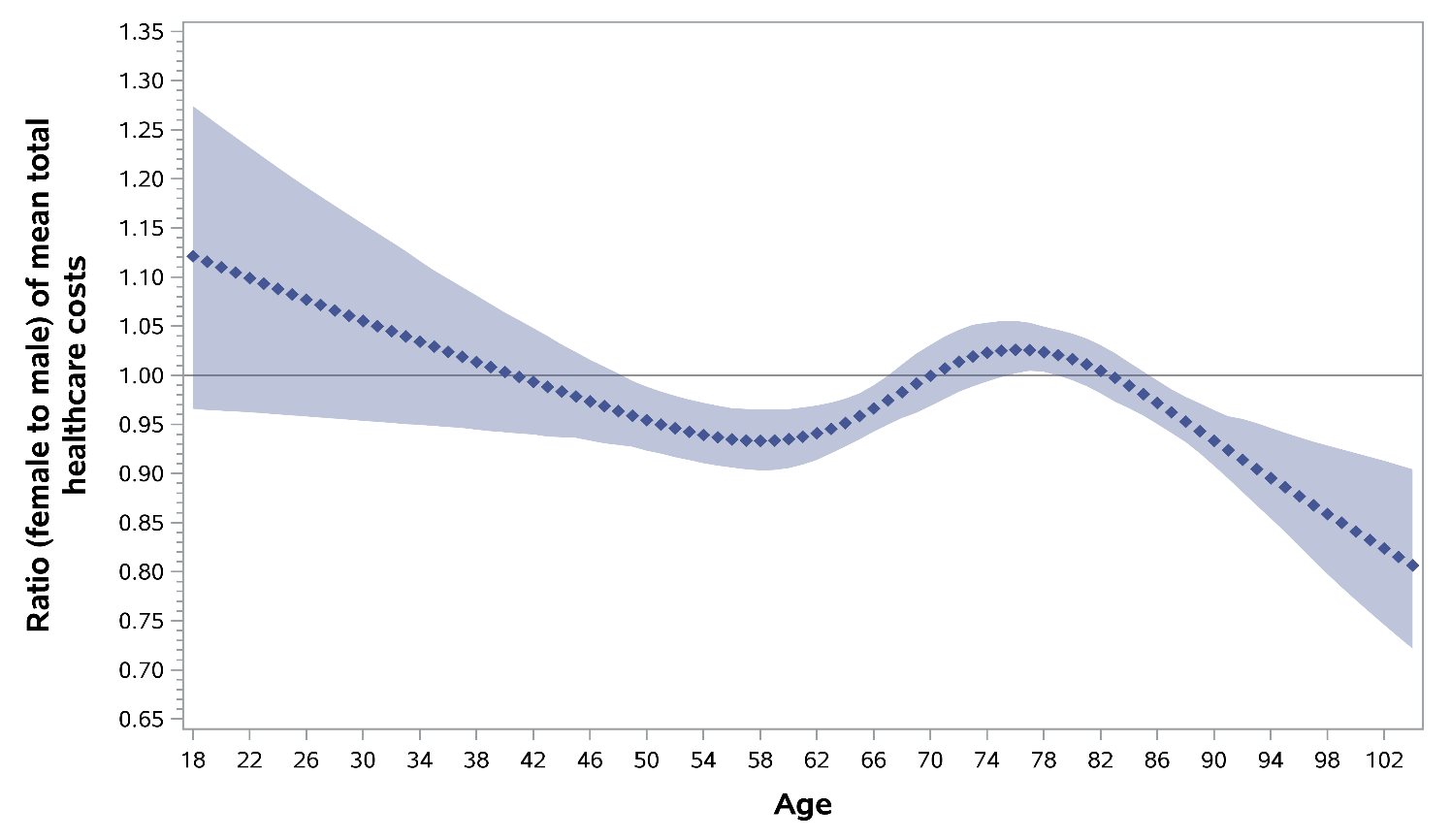

Supplement: Supplementary file 1 — Additional file 1. [file 12913_2021_6669_MOESM1_ESM.docx]
